# Differences in Clinical Manifestations of Acute and Early HIV-1 Infection between HIV-1 Subtypes in African Women

**DOI:** 10.1177/2325957413504827

**Published:** 2015-09

**Authors:** Tracy L. Lemonovich, Richard R. Watkins, Charles S. Morrison, Cynthia Kwok, Tsungai Chipato, Robert Musoke, Eric J. Arts, Immaculate Nankya, Robert A. Salata

**Affiliations:** 1 Division of Infectious Diseases and HIV Medicine, Case Western Reserve University, University Hospitals Case Medical Center, Cleveland, OH, USA; 2 Division of Infectious Diseases, Akron General Medical Center, Akron, OH, USA; 3 Family Health International, Durham, NC, USA; 4 Department of Obstetrics and Gynaecology, University of Zimbabwe Medical School, Harare, Zimbabwe; 5 Department of Medicine, Makerere University College of Health Sciences, Kampala, Uganda

**Keywords:** HIV-1 subtype, clinical manifestations, STDs, acute HIV infection, women

## Abstract

Little is known about the differences in clinical manifestations between women with various HIV-1 subtypes during acute (AI) and early (EI) HIV infection. In a longitudinal cohort study, clinical signs and symptoms among Uganda and Zimbabwe women with AI and EI were compared with HIV-negative controls; symptoms were assessed quarterly for 15 to 24 months. Early HIV infection was defined as the first visit during which a woman tested HIV antibody positive. Women who were HIV negative serologically but DNA polymerase chain reaction positive were considered AI. In all, 26 women were classified AI and 192 EI, with 654 HIV-negative controls. Primary HIV infection (AI and EI) was associated with unexplained fever (*P* <.01), weight loss (*P* <.01), fatigue (*P* <.01), inguinal adenopathy (*P* <.01), and cervical friability (*P* =.01). More women with subtype C infection had unexplained fever, fatigue, and abnormal vaginal discharge compared to subtype A or D infection. Inguinal adenopathy occurred less often in women with subtype A infection than those with subtype C or D infection.

## Introduction

The ability of HIV-1 to rapidly mutate in order to evade the human immune system has resulted in an extraordinary degree of viral diversity. At present, 3 groups of HIV-1 exist: major (M), outlier (O), and non-M and non-O (N).^1^ Group M is the predominant HIV-1 group worldwide, causing >90% of cases and is divided into subtypes A to K.^2^ In sub-Saharan Africa, nearly all subtypes are represented but their distribution is heterogeneous. In the southern regions of the continent, subtype C predominates while the central and eastern regions have a mix of subtypes A, C, D, and G along with recombinant viruses whose genomes result from recombination between subtypes within a dually infected host.^3,4^ In Uganda, subtypes A and D predominate,^5^ while HIV in Zimbabwe is almost exclusively of subtype C.^6^ Some studies have demonstrated differences in disease progression, immunogenicity, replication, and transmission among the different subtypes.^5,7–9^ In contrast, other reports have found no significant differences in disease progression between HIV-1 subtypes.^10,11^ The HIV-1 subtype does not seem to impact the response to combination antiretroviral therapy (cART), although more rapid viral load suppression is seen in subtypes C and A.^12^

A study from Seattle found that 89% of the patients reported symptoms with HIV seroconversion.^13^ These investigators noted that the 5 most common symptoms of primary HIV infection in their cohort were fever, sore throat, fatigue, weight loss, and myalgia. Among a cohort of female sex workers in Kenya, 76% reported at least 1 symptom consistent with acute HIV-1 infection (AI).^14^ The most common symptoms were fever (61%), headache (49%), fatigue (30%), arthralgia (30%), vomiting (20%), diarrhea (17%), pharyngitis (16%), rash (8%), and swollen glands (6%). Other studies have described a similar spectrum of symptoms with AI, including fever, fatigue, weight loss, rash, headache, lymphadenopathy, pharyngitis, and myalgias/arthralgias.^15,16^ Primary HIV-1 infection is associated with a high viral burden, which is a major factor in determining HIV transmission.^17^ Thus, it is important to identify those patients with AI or early infection (EI) so that HIV prevention counseling can be undertaken and clinical care can be initiated.

Between 1999 and 2004, when the data presented here were collected, Uganda and Zimbabwe were experiencing a generalized HIV-1 epidemic. As part of a large prospective cohort study in Zimbabwe and Uganda, women enrolled were routinely tested for a variety of reproductive tract infections (RTIs) as well as HIV. This article reports the differences identified in the clinical signs and symptoms of AI and EI between the HIV-1 subtypes in African women. In addition, we evaluated the effects of the clinical manifestations in AI and EI on the natural progression of HIV-1 during the follow-up period in this group of patients.

## Materials and Methods

The differences in clinical manifestations of HIV-1 in AI and EI between the different subtypes were determined through secondary analysis of data from the Hormonal Contraception and Risk of HIV Acquisition (HC-HIV) study. This cohort study examined the risk of acquisition of HIV infection and association with contraceptive type; detailed methods of the study have been published previously.^18^ The study was conducted between November 1999 and January 2004. It was approved by the ethical review committees of collaborating institutions in the United States, Uganda, and Zimbabwe, and all participating women provided written consent before participating in the study.

### Study Population

Zimbabwean and Ugandan women were recruited from urban and peri-urban family planning and mother–child health clinics.^18^ Because of lower than expected recruitment, enrollment in Uganda was expanded to populations at higher risk for HIV-1 infection, including commercial sex workers, military wives, and patients seen at sexually transmitted disease (STI) clinics that met enrollment criteria. At enrollment, participants were between the age of 18 and 35 years; were HIV uninfected, sexually active, and not pregnant; had not had a spontaneous or induced abortion in the past 3 months; and had not received a blood transfusion or injected drugs in the previous 3 months. Eligible women were using combined oral contraceptives, depot medroxyprogesterone acetate (DMPA), or a nonhormonal method of contraception (eg, condoms, withdrawal, sterilization). The number of women in each of these contraceptive groups was approximately equal at each enrollment site.^18^ Women were not eligible to participate if they had a hysterectomy or were using an intrauterine device.

### Data Collection Procedures

At their initial screening, consenting women were counseled and tested for HIV and *Treponema pallidum*. Women returned within 15 days for test results and possible enrollment. At enrollment, eligible participants were interviewed in their local language regarding demographics, sexual and contraceptive behavior, and reproductive health using structured questionnaires. They were counseled about HIV risk reduction and received condoms and contraceptives free of charge at each study visit. Women were specifically counseled regarding high-risk behaviors (multiple partners, unprotected sex, etc) and interactively discussed ways for risk reduction. A standardized physical and pelvic examination was performed, and specimens were collected to test for multiple RTIs. Participants were followed quarterly for 15 to 24 months and received a physical and pelvic examination, HIV testing, and a behavioral interview using a standardized questionnaire at each visit. This questionnaire was the basis for the data collected on symptoms in the study. A woman was considered to have EI at the first visit when she tested positive on an enzyme immune assay (EIA), with confirmatory testing by rapid test and Western blot. Specimens from the current and immediate prior visit were then tested by HIV polymerase chain reaction (PCR) to confirm the timing of infection. Women originally considered HIV negative by EIA on the immediate prior visit but later found to be DNA-PCR-positive at that visit were considered to have AI. The EI and AI groups were mutually exclusive, with women only contributing to 1 group. Three HIV-negative controls (NCs) were matched to each HIV-positive woman by country and study visit.

### Laboratory Methods

At enrollment, all participants were determined to be HIV negative by enzyme-linked immunosorbent assay (ELISA). At each quarterly visit, HIV testing was done by ELISA, with positive results confirmed by rapid testing, followed by Western blot and PCR testing. HIV DNA PCR results were the final arbiter of infection status. The date of HIV acquisition was defined as the date of the first positive PCR result, this being a reasonable approximation since the time between HIV acquisition and the first positive PCR result would be small in the context of this study.^18^

Polymerase chain reaction assays were conducted at each visit to detect *Chlamydia trachomatis* and *Neisseria gonorrhoeae *(Amplicor; Roche Diagnostics, Indianapolis, Indiana). Incident *N gonorrhoeae* and *C trichomatis* were defined as a positive PCR test after a negative test at the previous visit. Wet mount microscopy of vaginal fluid was conducted on-site to detect *Trichomonas vaginalis*, bacterial vaginosis (BV) by Amsel criteria, and vaginal yeast. Incident *T vaginalis* and vaginal yeasts were detected by presence on the wet mount after absence on the wet mount at the previous visit.

HIV plasma viral loads were determined at each study visit using the Roche Amplicor HIV-1 Monitor Test version 1.5 assay per the manufacturers’ protocol.^19^ To determine HIV-1 subtypes, DNA was extracted from whole blood using the Qiagen DNA extraction kit (Qiagen Inc, Gaithersberg, Maryland). The *env* gene was PCR amplified in the C2 to V3 region using an externally nested PCR amplification with primary pairs ENV B-ED14 (external) and ENV1-ENV2 (nested).^20^ The PCR products were purified using the Qiagen PCR purification kit and sequenced using the Beckman Coulter CEQ 8000 (Beckman Coulter, Inc, Brea, California) sequencer with the ENV1 forward primer. The sequences were analyzed and edited and are available in GenBank.

### Statistical Analysis

Differences in clinical signs and symptoms were examined using the Fisher exact test and chi-square test by HIV-1 infection (AI and EI versus HIV-negative controls), by country, by HIV-1 subtypes (A, C and D), and by AIDS end points (2 successive CD4 <200 cells/mm^3^ or World Health Organization advanced stage 3 or stage 4 disease). For women with AI or EI, clinical signs and symptoms were used at the study visit of the first positive HIV PCR result. Logistic regression was used to estimate the odds ratio and 95% confidence intervals for symptoms and signs among HIV-positive women compared with HIV-negative women. Multivariable analysis was performed for differences in clinical signs and symptoms in HIV-infected versus uninfected controls; all covariates with *P* values less than .05 from Fisher exact tests in the bivariate analysis were included in the multivariable analysis. A backward selection approach was used to obtain variables with *P* <.05 for inclusion in the final multivariable model. The odds ratios of the clinical signs and symptoms between HIV-positive and HIV-negative women in Uganda and Zimbabwe and among HIV-1 subtypes were tested for homogeneity using the Breslow-Day exact test. The Loess procedure was used to estimate the mean level of plasma viral load set points.^19^ The Wilcoxon Mann-Whitney test was used to test the hypothesis that viral load set point differed significantly between those patients with and those patients without signs and symptoms of HIV infection. Statistical analyses were performed using SAS (version 9.2; SAS Institute, Cary, North Carolina) and StatXact (version 9; Cytel Software Corporation, Cambridge, Massachusetts).

## Results

A total of 872 women were selected for the study, 218 HIV-infected women (155 from Zimbabwe and 63 from Uganda) and 654 HIV-negative controls (465 from Zimbabwe and 189 from Uganda). The study included 4 clinical study sites in Zimbabwe and 3 sites in Uganda. Two hundred fourteen HIV-infected women had HIV-1 subtype information available for analysis. The median age of study participants at enrollment was 25 years (range, 18-35 years), the median education was 10 years (range, 0-17 years), and the median number of live births was 2 (range, 0-10). Most participants, 748 (86%), lived with their primary sexual partner, and few women, 35 (4%), reported more than 1 sex partner in the 3 months before enrollment. The Ugandan cohort had a higher proportion of women who engaged in sexual risk behaviors than the Zimbabwean cohort, with 189 (75%) versus 559 (90%) women living with their primary sexual partner (*P* <.001) and 54 (21%) versus 15 (2%) with >1 sexual partner in the past 12 months (*P* <.001), respectively. Ugandan women were also more likely to report a history of STIs and RTI symptoms in the previous 12 months. Circumcision of their primary male partner was reported by 91 (36%) participants from Uganda and 56 (9%) participants from Zimbabwe. HIV prevalence at screening was 39% in Zimbabwe and 17% in Uganda.

Participants with AI and EI were more likely than HIV-negative controls to have symptoms of unexplained fever, weight loss, fatigue, diarrhea, swollen glands, headaches, abnormal vaginal discharge, and abdominal pain (Table 1). Clinical signs more common in the AI and EI groups included inguinal adenopathy, cervical friability, trichomonas, BV, gonorrhea, and chlamydia. In multivariable analyses, we found unexplained fever, weight loss, fatigue, inguinal adenopathy, cervical friability, BV, and gonorrhea were associated with AI and EI compared with HIV-uninfected controls.

**Table 1.** Clinical Symptoms and Signs and STI among Women with Acute (AI) and Early (EI) versus HIV-NCs.

Characteristic	AI (n = 26), n (%)	EI (n = 192), n (%)	NC (n = 654), n (%)	*P* Value^a^	OR (95% CI)^b^ HIV+ versus HIV−	Multivariable OR (95% CI)^c^ HIV+ versus HIV−
Symptoms^d^					
Unexplained fever	5 (19.23)	19 (9.9)	20 (3.06)	<.0001	3.9 (2.1-7.3)	2.7 (1.2-6.4)
Night sweats	2 (7.69)	6 (3.13)	16 (2.45)	.1974	1.5 (0.6-3.6)	
Weight loss	1 (3.85)	21 (10.94)	7 (1.07)	<.0001	10.4 (4.4-24.6)	8.5 (2.5-28.9)
Fatigue	2 (7.69)	17 (8.85)	9 (1.38)	<.0001	6.8 (3-15.4)	3.4 (1.3-9.2)
Diarrhea	0 (0)	4 (2.08)	1 (0.15)	.0223	12.2 (1.4-109.5)	
Yeast	0 (0)	18 (9.38)	33 (5.05)	.0455	1.7 (0.9-3.1)	
Swollen glands	0 (0)	3 (1.56)	0 (0)	.0174	N/A	
Unexplained severe rash	0 (0)	5 (2.6)	10 (1.53)	.5889	1.5 (0.5-4.5)	
Headaches	5 (19.23)	29 (15.1)	59 (9.02)	.0157	1.9 (1.2-2.9)	
Nausea	3 (11.54)	13 (6.77)	30 (4.59)	.1270	1.6 (0.9-3.1)	
Abnormal vaginal discharge	1 (3.85)	28 (14.58)	30 (4.59)	<.0001	3.2 (1.9-5.5)	
Genital itching	3 (11.54)	32 (16.67)	71 (10.86)	.0955	1.6 (1-2.4)	
Abdominal pain	5 (19.23)	13 (6.77)	33 (5.05)	.0163	1.7 (0.9-3.1)	
Pain during sex	1 (3.85)	10 (5.21)	23 (3.52)	.4861	1.5 (0.7-3)	
Genital warts	1 (3.85)	1 (0.52)	2 (0.31)	.0978	3 (0.4-21.5)	
Signs						
Inguinal adenopathy	3 (11.54)	51 (26.98)	46 (7.13)	<.0001	4.4 (2.8-6.7)	4.6 (2.8-7.6)
Abnormal vaginal epithelium	23 (95.83)	179 (95.72)	623 (97.8)	.1730	0.5 (0.2-1.2)	
Strawberry cervix	1 (4)	4 (2.16)	10 (1.57)	.3611	1.5 (0.5-4.5)	
Cervical friability	9 (45)	89 (51.15)	226 (38.44)	.0114	1.6 (1.2-2.3)	1.7 (1.2-2.5)
Candida	3 (11.54)	27 (14.29)	69 (10.73)	.3663	1.3 (0.9-2.1)	
Genital ulcers	1 (3.85)	1 (0.53)	6 (0.93)	.2831	1 (0.2-5)	
Trichomonas (wet mount)	1 (3.85)	8 (4.35)	7 (1.1)	.0139	4 (1.5-11)	
Bacterial vaginosis (Amsel)	5 (19.23)	62 (32.8)	95 (14.77)	<.0001	2.6 (1.8-3.7)	2.1 (1.4-3.2)
Gonorrhea (PCR)	4 (15.38)	24 (12.9)	6 (0.94)	<.0001	16 (6.5-39.2)	14.9 (5.4-41.0)
Chlamydia (PCR)	2 (7.69)	14 (7.53)	11 (1.73)	.0003	4.6 (2.1-10.2)	

Abbreviations: AI, acute HIV infection; CI, confidence interval; EI, early HIV infection; N/A, not available; NCs, negative controls; OR, odds ratio; PCR, polymerase chain reaction; STI, sexually transmitted infection

^a^ From Fisher exact test comparing AI + EI versus NCs.

^b^ The OR and 95% CI for HIV-infected women with AI and EI combined versus HIV-seronegative women.

^c^ Using backward selection approach to obtain variables in the final multivariable model.

^d^ Reported symptoms since the last study visit.

We sought to determine whether there were significant differences in clinical signs and symptoms of AI and EI HIV-1 infection between women in Uganda and those in Zimbabwe (Table 2). Compared to HIV-negative controls, unexplained fever, weight loss, fatigue, yeast, headache, abnormal vaginal discharge, and inguinal adenopathy were more common in Zimababwe and cervical friability was more common in Uganda. We detected heterogeneity between the 2 countries at the *P* <.05 significance level for abnormal vaginal discharge. There was weaker evidence of possible heterogeneity (.05 < *P* <.10) for unexplained fever, weight loss, and headache.

**Table 2.** Clinical Symptoms and Signs for HIV Status by Country.

	Uganda			Zimbabwe			*P* Value^a ^for Testing Homogeneity of ORs between Uganda and Zimbabwe
Characteristic	HIV+ (n = 63), n (%)	HIV− (n = 189), n (%)	OR (95% CI) HIV+ versus HIV−	HIV+ (n = 155), n (%)	HIV− (n = 465), n (%)	OR (95% CI) HIV+ versus HIV−	
Symptoms^b^							
Unexplained fever	13 (20.63)	17 (8.99)	2.63 (1.09-6.18)	11 (7.1)	3 (0.65)	11.76 (3.04-66.32)	.09
Weight loss	7 (11.11)	5 (2.65)	4.60 (1.20-19.0)	15 (9.68)	2 (0.43)	24.80 (5.63-224.8)	.09
Fatigue	3 (4.76)	4 (2.12)	2.31 (0.32-14.04)	16 (10.32)	5 (1.08)	9.88 (3.37-34.92)	.16
Diarrhea	1 (1.59)	1 (0.53)	3.03 (0.04-239.3)	3 (1.94)	0 (0)	NA	.40
Yeast	2 (3.17)	10 (5.29)	0.59 (0.06-2.87)	16 (10.32)	23 (4.95)	2.12 (1.06-4.51)	.16
Swollen glands	0 (0)	0 (0)	NA	3 (1.94)	0 (0)	NA	NA
Headaches	3 (4.76)	14 (7.41)	0.65 (0.11-2.35)	31 (20)	45 (9.68)	2.33 (1.36-3.95)	.08
Abnormal vaginal discharge	7 (11.11)	18 (9.52)	1.19 (0.40-3.17)	22 (14.19)	12 (2.58)	6.24 (2.86-14.18)	.01
Genital itching	16 (25.4)	39 (20.63)	1.32 (0.62-2.66)	19 (12.26)	32 (6.88)	1.89 (0.98-3.56)	.49
Abdominal pain	7 (11.11)	18 (9.52)	1.19 (0.40-3.17)	11 (7.1)	15 (3.23)	2.29 (0.93-5.47)	.36
Genital warts	1 (1.59)	1 (0.53)	3.03 (0.04-239.3)	1 (0.65)	1 (0.22)	3.01 (0.04-237.0)	1.00
Signs^b^							
Inguinal adenopathy	9 (15)	12 (6.56)	2.46 (0.86-6.73)	45 (29.03)	34 (7.36)	5.19 (3.08-8.76)	.20
Cervical friability	20 (38.46)	31 (18.56)	2.37 (1.16-4.77)	78 (54.93)	195 (46.32)	1.40 (0.96-2.05)	.18

Abbreviations: CI, confidence interval; N/A, not available; OR, odds ratio.

^a^ From Breslow-Day exact test.

^b^ Reported symptoms/signs since the last study visit.

Clinical signs and symptoms were characterized by HIV subtype (Table 3). The 3 HIV-1 subtypes identified among the study participants were A, C, and D. All women with HIV-1 subtypes A and D were from Uganda, whereas all but 1 participant with subtype C were from Zimbabwe. Subtype C was found in 156 women, subtype A was found in 39 women, and subtype D was found in 19 women. Unexplained fever, fatigue, and abnormal vaginal discharge were seen more commonly among subtype C infection than its matched controls with subtypes A and D infections. Inguinal adenopathy occurred less often among women with subtype A infection than its matched controls with subtypes C and D, where it was seen much more commonly among HIV-infected women than among HIV-negative women.

**Table 3.** Clinical Symptoms and Signs and STI among Women with Subtypes^a^ Versus Controls.^b^

	Subtype A			Subtype C			Subtype D			*P* Value^d ^for Testing Homogeneity of ORs among Subtypes
Characteristic	Case (n = 39), n (%)	Control (n = 125), n (%)	OR (95% CI) Case versus Control	Case^c^ (n = 156), n (%)	Control (n = 468), n (%)	OR (95% CI) Case versus Control	Case (n = 19), n (%)	Control (n = 61), n (%)	OR (95% CI) Case versus Control	
Symptoms^e^										
Unexplained fever	6 (15.38)	9 (7.2)	2.3 (0.8-7.1)	12 (7.69)	3 (0.64)	12.9 (3.6,46.4)	5 (26.32)	8 (13.11)	2.4 (0.7,8.4)	.08
Weight loss	3 (7.69)	4 (3.2)	2.5 (0.5-11.8)	15 (9.62)	3 (0.64)	16.5 (4.7,57.8)	2 (10.53)	0 (0)	N/A	.17
Fatigue	1 (2.56)	4 (3.2)	0.8 (0.1-7.3)	16 (10.26)	5 (1.07)	10.6 (3.8,29.4)	2 (10.53)	0 (0)	N/A	.05
Diarrhea	0 (0)	0 (0)	N/A	3 (1.92)	0 (0)	N/A	1 (5.26)	1 (1.64)	3.3 (0.2-56)	.43
Yeast	1 (2.56)	5 (4)	0.6 (0.1-5.6)	16 (10.26)	23 (4.91)	2.2 (1.1,4.3)	1 (5.26)	5 (8.2)	0.6 (0.1-5.7)	.38
Swollen glands	0 (0)	0 (0)	N/A	3 (1.92)	0 (0)	N/A	0 (0)	0 (0)	N/A	N/A
Headaches	2 (5.13)	11 (8.8)	0.6 (0.1-2.6)	31 (19.87)	45 (9.62)	2.3 (1.4,3.8)	1 (5.26)	3 (4.92)	1.1 (0.1-11)	.17
Abnormal vaginal discharge	5 (12.82)	13 (10.4)	1.3 (0.4-3.8)	22 (14.1)	13 (2.78)	5.7 (2.8,11.7)	2 (10.53)	4 (6.56)	1.7 (0.3-10)	.05
Genital itching	9 (23.08)	26 (20.8)	1.1 (0.5-2.7)	19 (12.18)	32 (6.84)	1.9 (1.0,3.4)	6 (31.58)	13 (21.31)	1.7 (0.5-5.4)	.66
Abdominal pain	5 (12.82)	12 (9.6)	1.4 (0.5-4.2)	11 (7.05)	15 (3.21)	2.3 (1.0,5.1)	2 (10.53)	6 (9.84)	1.1 (0.2-5.8)	.70
Genital warts	1 (2.56)	1 (0.8)	3.3 (0.2-53.4)	1 (0.64)	1 (0.21)	3 (0.2,48.4)	0 (0)	0 (0)	N/A	1.0
Signs										
Inguinal adenopathy	3 (8.11)	9 (7.5)	1.1 (0.3-4.2)	46 (29.49)	34 (7.31)	5.3 (3.2,8.7)	5 (26.32)	3 (5)	6.8 (1.4-31.8)	.06
Cervical friability	13 (38.24)	24 (21.82)	2.2 (1.0-5.1)	78 (54.55)	195 (46.1)	1.4 (1.0,2.1)	6 (42.86)	7 (12.73)	5.1 (1.4-19.3)	.25

Abbreviations: CI, confidence interval; N/A, not available; OR, odds ratio; STI, sexually transmitted infection.

^a^ Four Ugandan women were excluded as their subtype data were not available.

^b^ Program run on August 7, 2012, and data frozen in September 2006.

^c^ One Ugandan woman had subtype C.

^d^ From Breslow-Day exact test.

^e^ Reported symptoms since the last study visit.

We also investigated whether clinical signs and symptoms during EI and AI were associated with viral load set point and HIV disease progression. A higher viral load set point (log_10_ plasma viral load) was associated with weight loss (median 4.83 versus 4.29) and inguinal adenopathy (median 4.69 versus 4.25; Table 4). None of the clinical signs and symptoms we evaluated were associated with progression to our primary end point of AIDS diagnosis or to our secondary end points of AIDS diagnosis, death, or initiation of combined antiretroviral therapy (cART) during the follow-up period (Table 5).

**Table 4.** Viral Load at Set Point by Clinical Symptoms and Signs.

Characteristics	Log_10_ Plasma VL		*P* Value^a^
	With Symptoms and Signs	Without Symptoms and Signs	
Symptoms^b^			
Unexplained fever			
Mean (SD)	4.5 (0.83)	4.2 (0.98)	
Median (Q1-Q3)	4.67 (3.67-5.2)	4.3 (3.6-4.95)	.1817
Weight loss			
Mean (SD)	4.68 (0.89)	4.19 (0.96)	
Median (Q1-Q3)	4.83 (3.72-5.39)	4.29 (3.6-4.95)	.0346
Fatigue			
Mean (SD)	4.36 (0.68)	4.22 (0.99)	
Median (Q1-Q3)	4.35 (4.06-4.69)	4.33 (3.58-5)	.8436
Diarrhea			
Mean (SD)	4.33 (1.56)	4.23 (0.96)	
Median (Q1-Q3)	4.59 (2.66-5.74)	4.33 (3.61-4.99)	.8206
Yeast			
Mean (SD)	4.14 (1.07)	4.24 (0.96)	
Median (Q1-Q3)	4.18 (3.31-4.9)	4.33 (3.65-5)	.6977
Swollen glands			
Mean (SD)	4.1 (0.77)	4.24 (0.97)	
Median (Q1-Q3)	4.1 (3.33-4.87)	4.33 (3.61-5)	.6650
Headaches			
Mean (SD)	4.3 (0.81)	4.22 (0.99)	
Median (Q1-Q3)	4.33 (3.66-5.04)	4.33 (3.61-4.98)	.9035
Abnormal vaginal discharge			
Mean (SD)	4.35 (0.78)	4.22 (0.99)	
Median (Q1-Q3)	4.66 (3.74-4.92)	4.3 (3.6-5.01)	.6623
Genital itching			
Mean (SD)	4.15 (0.86)	4.25 (0.99)	
Median (Q1-Q3)	4.22 (3.6-4.87)	4.33 (3.61-5.03)	.4551
Abdominal pain			
Mean (SD)	4.44 (0.74)	4.21 (0.98)	
Median (Q1-Q3)	4.73 (3.75-4.82)	4.3 (3.6-5)	.4365
Genital warts			
Mean (SD)	2.94 (N/A)	4.24 (0.96)	
Median (Q1-Q3)	2.94 (2.94-2.94)	4.33 (3.61-5)	.1713
Signs^b^			
Inguinal adenopathy			
Mean (SD)	4.52 (0.86)	4.14 (0.98)	
Median (Q1-Q3)	4.69 (4.1-5.2)	4.25 (3.49-4.92)	.0095
Cervical friability			
Mean (SD)	4.15 (0.98)	4.29 (0.97)	
Median (Q1-Q3)	4.2 (3.56-4.96)	4.57 (3.75-5)	.2664

Abbreviations: SD, standard deviation; VL, viral load.

^a ^Wilcoxon Mann-Whitney test to explore the VL differences among yes/no groups.

^b ^Reported at seroconversion visit.

**Table 5.** Clinical Symptoms and Signs by Primary End Point.^a^

Characteristic	Had Primary End Point (n = 86), n (%)	Without Primary End Point (n = 107), n (%)	*P* Value^b^	OR (95% CI) Had versus Without
Symptoms^c^				
Unexplained fever	11 (12.79)	11 (10.28)	.6517	1.3 (0.5-3.1)
Weight loss	7 (8.14)	11 (10.28)	.8041	0.8 (0.3-2.1)
Fatigue	10 (11.63)	8 (7.48)	.3335	1.6 (0.6-4.3)
Diarrhea	0 (0)	3 (2.8)	.2550	N/A
Yeast	5 (5.81)	11 (10.28)	.3039	0.5 (0.2-1.6)
Swollen glands	1 (1.16)	2 (1.87)	1.0000	0.6 (0.1-6.9)
Headaches	14 (16.28)	15 (14.02)	.6894	1.2 (0.5-2.6)
Abnormal vaginal discharge	8 (9.3)	18 (16.82)	.1429	0.5 (0.2-1.2)
Genital itching	11 (12.79)	21 (19.63)	.2448	0.6 (0.3-1.3)
Abdominal pain	5 (5.81)	12 (11.21)	.2120	0.5 (0.2-1.4)
Genital warts	0 (0)	1 (0.93)	1.0000	N/A
Signs^c^				
Inguinal adenopathy	18 (21.43)	28 (26.17)	.4978	0.8 (0.4-1.5)
Cervical friability	37 (50.68)	50 (51.02)	1.0000	1 (0.5-1.8)

Abbreviations: CI, confidence interval; N/A, not available; OR, odds ratio.

^a^ Defined as a participant with AIDS diagnosis. Missing 25 end points as HIV+ women did not enroll into Hormonal Contraception and Risk of HIV-Acquisition (HC-HIV) study.

^b^ From Fisher exact test.

^c^ Reported at seroconversion visit.

## Discussion

We observed a number of differences in the clinical signs and symptoms of EI and AI between 3 distinct subtypes. Prior studies have suggested that subtype D may be more pathogenic than subtypes A and C, possibly due to a higher replicative capacity (viral fitness).^3,5,21–24^ Few studies have evaluated the differences in initial clinical presentation of AI between the HIV-1 subtypes.^25^ Our study shows some important differences in initial signs and symptoms between the subtypes, particularly with subtype C where unexplained fever, fatigue, and abnormal vaginal discharge were more common than its matched controls with the other subtypes. Nevertheless, the absolute levels (prevalence) of some of these symptoms, such as unexplained fever, were still higher among women with subtype D infection. These signs and symptoms may be reflective of the pathogenicity of the specific subtypes. Specifically, subtype D infection may be more often associated with fever due to increased viral fitness and therefore more inflammatory response in these patients.^23,26,27^ Interestingly, however, none of the initial clinical manifestations were associated with progression of HIV to an AIDS diagnosis, death, or initiation of cART during the follow-up period. Our study focused on women, hence our emphasis on RTIs and including such symptoms as genital itching. Our data on the clinical manifestations of AI and EI are similar to those previously reported from other resource-limited settings. Lavreys et al also found that fever, headache, and inguinal adenopathy were common in women who seroconverted to HIV-1.^28^

Our study has a number of strengths. First, it includes women with EI and AI with known timing of HIV acquisition. This timing was determined by PCR-based methods and therefore was more precise than serology. Second, we had a large number of women with EI and AI with multiple subtypes. Third, the study was prospective with quarterly evaluations that allow for earlier detection of clinical signs and symptoms associated with HIV infection. Finally, we enrolled women from 2 different sub-Saharan countries, which make our results more generalizable than if the study was conducted on a selected high-risk population (eg, commercial sex workers) from a limited geographical area.

There are also some limitations to our study. We sequenced only the C2 to V3 region of *env* and could not fully explore the issue of recombinant viral strains. Although subtype D has been shown in some studies to have a faster rate of progression,^5–7^ our sample size of 19 women with subtype D was small and may have precluded finding significant differences in HIV disease progression in women presenting with specific clinical manifestations in this group. Additionally, since HIV subtypes were exclusive to either Zimbabwe or Uganda, there could be a reporting bias in regard to collection of the clinical signs and symptoms by country. However, serial training in collection of clinical information was performed for the duration of the study in both the countries, and clinical events were carefully vetted on-site and by the study clinical consultant, hopefully minimizing this bias. Some RTI episodes may have gone unnoticed because of the 3-month interval between study visits. Finally, we relied on self-reported symptoms including headaches, fatigue, abnormal vaginal discharge, abdominal pain, swollen glands, genital itching, and diarrhea, the accuracy of which is unknown.

In summary, this large, multisite study found specific initial clinical manifestations of AI and EI compared with HIV uninfected women as well as particular manifestations associated with subtypes C (fever, fatigue, abnormal vaginal discharge, and inguinal adenopathy) and D (inguinal adenopathy) infections. These clinical manifestations were not associated with the subsequent progression of HIV disease. Larger studies are needed to determine whether these differences in initial clinical signs and symptoms in the different subtypes may be associated with differences in HIV outcomes. Early recognition of these potential symptoms and signs of primary HIV infection are important for initiation of HIV testing, leading to entry into clinical care and HIV risk reduction counseling.^29^ The importance of early HIV diagnosis from a public health standpoint cannot be overstated, both to prevent further HIV-related complications in the infected individual and to decrease transmission to sexual partners. The diagnosis of AI and/or EI allows for greater opportunities for prevention of HIV transmission via behavior risk reduction, particularly during the time period when transmission risk is potentially greatest due to high viral loads. In particular, certain signs and symptoms in our study such as weight loss and inguinal adenopathy were associated with higher viral load set points and therefore increased potential for HIV transmission. Transmission to uninfected sexual partners could also be reduced by earlier initiation of cART.^30^ Overall, the recognition of the signs and symptoms of primary HIV infection in this and other patient populations has significant potential implications.
